# PAC-5 Gene Expression Signature for Predicting Prognosis of Patients with Pancreatic Adenocarcinoma

**DOI:** 10.3390/cancers11111749

**Published:** 2019-11-07

**Authors:** Jieun Kim, Yong Hwa Jo, Miran Jang, Ngoc Ngo Yen Nguyen, Hyeong Rok Yun, Seok Hoon Ko, Yoonhwa Shin, Ju-Seog Lee, Insug Kang, Joohun Ha, Tae Gyu Choi, Sung Soo Kim

**Affiliations:** 1 Department of Biomedical Science, Graduate School, Kyung Hee University, Seoul 02447, Korea; popje124@khu.ac.kr (J.K.); kimruby@khu.ac.kr (N.N.Y.N.); foryou018@khu.ac.kr (H.R.Y.); jac03032@khu.ac.kr (Y.S.); iskang@khu.ac.kr (I.K.); hajh@khu.ac.kr (J.H.); 2 Biomedical Science Institute, Kyung Hee University, Seoul 02447, Korea; yonghwa.jo@khu.ac.kr (Y.H.J.); mrjang421@khu.ac.kr (M.J.); 3 Department of Biochemistry and Molecular Biology, School of Medicine, Kyung Hee University, Seoul 02447, Korea; 4 Department of Emergency Medicine, School of Medicine, Kyung Hee University, Seoul 02447, Korea; 5 Department of Systems Biology, University of Texas MD Anderson Cancer Center, Houston, TX 77030, USA; jlee@mdanderson.org

**Keywords:** pancreatic adenocarcinoma, gene expression signature, prognostic prediction, adjuvant therapies

## Abstract

Pancreatic adenocarcinoma (PAC) is one of the most aggressive malignancies. Intratumoural molecular heterogeneity impedes improvement of the overall survival rate. Current pathological staging system is not sufficient to accurately predict prognostic outcomes. Thus, accurate prognostic model for patient survival and treatment decision is demanded. Using differentially expressed gene analysis between normal pancreas and PAC tissues, the cancer-specific genes were identified. A prognostic gene expression model was computed by LASSO regression analysis. The PAC-5 signature (LAMA3, E2F7, IFI44, SLC12A2, and LRIG1) that had significant prognostic value in the overall dataset was established, independently of the pathological stage. We provided evidence that the PAC-5 signature further refined the selection of the PAC patients who might benefit from postoperative therapies. SLC12A2 and LRIG1 interacted with the proteins that were implicated in resistance of EGFR kinase inhibitor. DNA methylation was significantly involved in the gene regulations of the PAC-5 signature. The PAC-5 signature provides new possibilities for improving the personalised therapeutic strategies. We suggest that the PAC-5 genes might be potential drug targets for PAC.

## 1. Introduction

Pancreatic cancer is an intractable malignancy, which is the fourth-leading cause of cancer deaths in the United States, with 56,770 new cases and 45,750 deaths in 2019 [1]. It constitutes a small percentage of all cancer deaths (7.2%). However, it is one of the fatal types of cancers with a five-year survival rate of only 9%. The vast majority of pancreatic cancers (>85%) are adenocarcinomas occurring in exocrine glands of the pancreas. Most of pancreatic adenocarcinoma (PAC) patients typically present advanced stages at the diagnosis. Surgery is considered the most effective treatment and the only therapeutic intervention, but only 20% of the patients are eligible for resection [2].

The American Joint Committee on Cancer (AJCC) staging system has been widely applied worldwide to provide guidelines for prognostic assessment and therapeutic decisions in PAC. The AJCC staging system is based on three components: size and/or local extent of the primary tumour (T), the involvement of regional lymph nodes (N), and metastasis (M). However, it is unable to describe tumour behaviour comprehensively. Indeed, PAC patients with the same AJCC stage may have different clinical prognosis after receiving the same treatment [3]. Thus, authorised model should be further proposed to complement the current pathological staging.

In PAC patients, gemcitabine is still employed as the baseline agent for adjuvant chemotherapy [2]. Thereafter, a combination of gemcitabine with FOLFIRINOX [4] or albumin-bound paclitaxel (nab-paclitaxel) [5] have become first-line therapies. However, the majority of patients poorly respond to these chemotherapeutic agents, and the therapeutic failure rather accelerates drug-resistance and metastatic progression [6]. This phenomenon is supported by intratumoural molecular heterogeneity that arises at multiple stages during tumour progression [7]. Tumourigenesis of PAC involves mutual interactions of diverse factors, including gene mutations and microenvironmental conditions [8]. Furthermore, tumour heterogeneity is closely associated with therapeutic sensitivity [7,9]. It is therefore vital to understand the underlying mechanisms in order to increase the treatment efficacy and improve patient outcomes. 

With the remarkable advances in bioinformatic technologies, prognostic gene expression signatures have extensively been developed, which reflect various clinicopathological and demographic factors. To date, commercial gene signatures were successfully established to predict prognosis and help therapeutic decision in various cancer patients such as head and neck [10] and breast [11]. In PAC, several previous studies have attempted to develop tumour subtype for prediction of prognosis [12,13,14,15] or therapeutic benefit [16]. However, clinical applications are not yet available. Thus, it is necessary to develop molecular classifier that allows to accurately predict the prognosis of the individual patient via the understanding of tumour heterogeneity in PAC. Furthermore, a good molecular classifier that minimises harmfulness from the overtreatment of patients and thus provides therapeutic benefit in safe is required.

Here, we established a novel molecular classifier that accurately predicted the prognosis of the PAC patients, which was closely associated with tumour-specific gene expression. The PAC-5 gene expression signature would give benefit to PAC patients by selecting patients who were suitable for adjuvant therapies. Further, we attempted to provide possibilities for improving prognostic models of PAC heterogeneity via extensive analyses.

## 2. Results

### 2.1. Establishment of a Prognostic Gene Expression Signature

**Figure 1:**
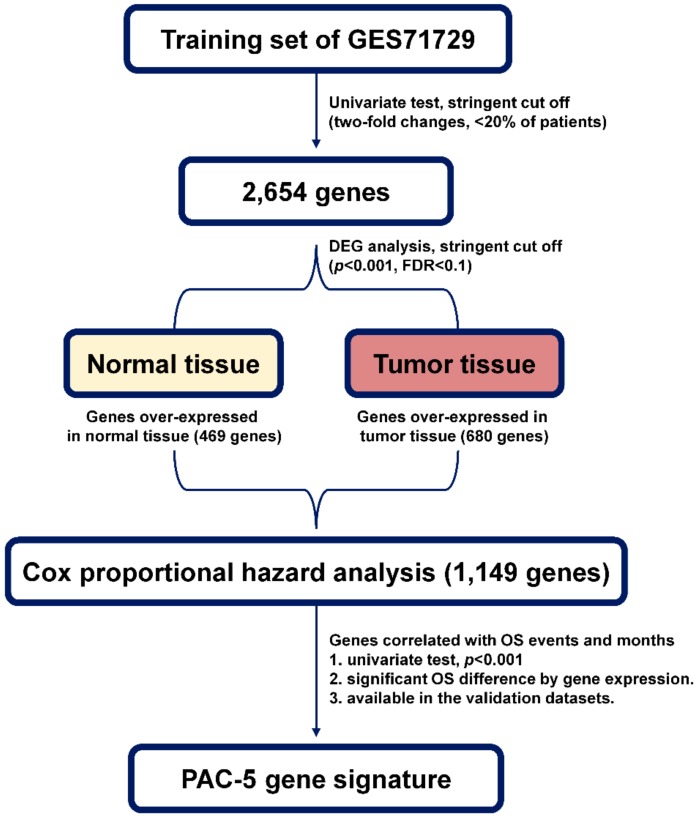
Schematic overview of the strategy used for prognostic model construction.

**Figure 2:**
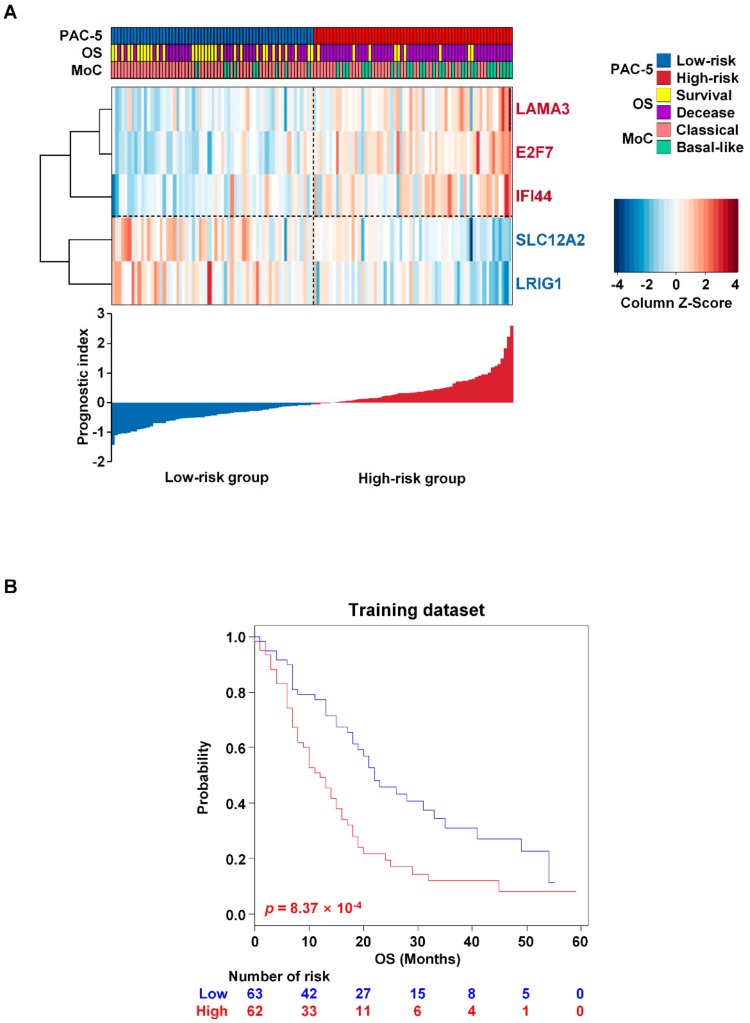
Establishment of the PAC-5 signature. (**A**) Survival and clinical information were associated with the heatmap of the two risk-groups in the training dataset (upper panel). The gene expression score colour keys were presented in the legends, with red indicating higher expression and blue lower expression. The patients were also clustered into two groups (classic and basal-like), based on the Moffitt classification. MoC, Moffitt classification. The prognostic index for each patient was calculated according to the weight of each gene (lower histogram). (**B**) Kaplan–Meier plots for OS of two risk-groups in the training dataset. *p*-Values were computed by log-rank test.

In order to generate a molecular classifier that distinguishes PAC patients into low- and high-risk groups, the gene expression data have been examined in relation to survival information. GSE71729 was used as a training dataset. A flow chart of the procedure used to generate the gene signature was provided in Figure 1. Initially, 2654 genes were obtained through filtering gene set intensity. Then, differentially expression genes (DEGs) analysis between normal pancreas and tumour tissues was employed to identify cancer-related genes, by which 1149 tumour-specific genes were obtained (Table S2). These genes were used for least absolute shrinkage and selection operator (LASSO) regression analysis with overall survival (OS) as the survival endpoint. As a result, we obtained a subset of prognostic genes: *LAMA3*, *E2F7*, *IFI44*, *SLC12A2*, *LRIG1*, *DUOXA1*, and *RBM1*. However, *DUOXA1* expression did not act as an independent prognostic biomarker to stratify the patients into distinct risk-groups, and the gene expression data for *RBM1* were not available in the external validation datasets. These two genes were excluded from establishing the final gene expression signature related to OS. Thus, we established a prognostic model that was termed the PAC-5 signature, including five genes (*LAMA3*, *E2F7*, *IFI44*, *SLC12A2*, and *LRIG1*, Table S3). Based on the PAC-5 gene expression patterns, the low- and high-risk groups were accurately represented by two clusters (Figure 2A, upper panel). To confirm whether the PAC-5 genes were tumour-specifically expressed, the mRNA expression levels of five genes between normal subjects and risk-groups of the PAC patients were evaluated. The expression levels of *LAMA3*, *E2F7*, *IFI44*, and *SLC12A2* were higher in PACs than in the normal pancreases. However, *LRIG1* mRNA was less expressed in PACs than in the normal pancreatic tissues. In the analysis between the tumour tissues, mRNAs of *LAMA3*, *E2F7*, and *IFI44* were more expressed in the tissues of the high-risk group than in those of low-risk group, whereas the expression levels of *IFI44* and *LRIG1* were lower in the high-risk group than in low-risk group (Figure S1A–E). Prognostic index values were calculated based on the PAC-5 signature for all patients and normal subjects. The patients were classified into low- (*n* = 63) and high-risk (*n* = 73) groups by their prognostic indices (Figure 2A, lower histogram). Prognostic index values for the high-risk group were significantly higher than those for the two other groups (Figure S1F).

Moffitt et al. (GSE71729 training dataset) [13] previously suggested two distinctive subtypes for predicting prognosis of PAC patients, of which a ‘basal-like subtype’ was associated with poorer prognostic outcome than a ‘classical subtype’. These Moffitt classification subtypes were associated with the gene expression patterns and histological cellularity in PACs. Interestingly, LAMA3 was also used for their genes in subtype-discrimination, of which over-expression was related to the ‘basal-like subtype’. Thus, to further evaluate the relevance between PAC-5 signature and Moffitt classification, we performed an association analysis using χ^2^ test; the risk-groups by the PAC-5 signature were significantly correlated with the Moffitt classification (*p* = 1.12 × 10^−3^, Table S4). To further verify the survival difference between low- and high-risk groups in the training dataset, we employed the Kaplan–Meier survival curve analysis. As a result, the Kaplan–Meier plot indicated a significant prognostic difference between the low- and high-risk groups at a median OS of 27.4 and 13.7 months, respectively (*p* = 8.37 × 10^−4^, Figure 2B).

### 2.2. Survival Analysis and Clinical Relevance of PAC-5 Signature in the Validation Datasets

**Figure 3:**
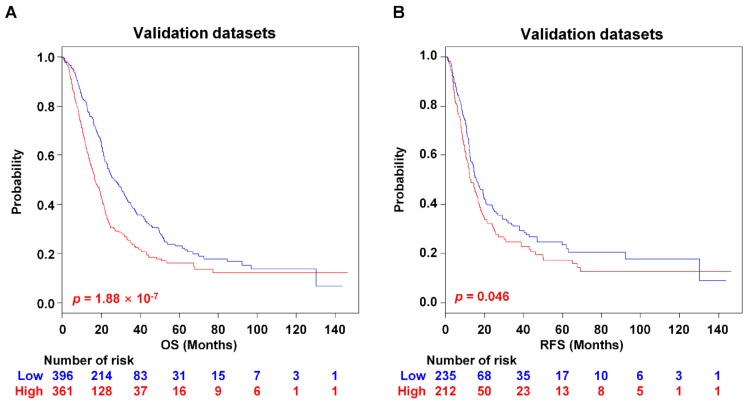
Kaplan–Meier survival analysis of the PAC-5 signature in validation datasets. (**A**,**B**) Kaplan–Meier survival plots for OS and RFS of two risk-groups in the validation datasets. The *p*-Values were computed by the log-rank test.

Next, to further estimate the robustness of the classifier, the PAC-5 signature was validated in the combined five microarray or three RNA-seq datasets. During (leave-one-out cross-validation LOOCV, the specificity and the sensitivity for correctly predicting risk were 0.839 and 0.905 in compound covariate predictor, respectively. The PAC-5 signature significantly classified patients into low- and high-risk groups at median OS of 30.4 and 17.7 months in the combined validation datasets (*p* = 1.88 × 10^−7^, Figure 3A), and RFS of 17.5 and 15.5 months in the combined validation datasets (*p* = 0.046, Figure 3B). Kaplan–Meier plots also showed significant prognostic differences in the microarray datasets and RNA-seq datasets (*p* = 4.87 × 10^−3^ and *p* = 6.94 × 10^−7^ for OS, respectively, Figure S2A,B).

One external dataset, GSE62452, had gene expression data of adjacent tissues paired to the data of their tumour tissues (*n* = 61). To further intensify that the PAC-5 genes were tumour-specifically expressed, we evaluated the mRNA expression levels of five genes between adjacent tissues and their tumour tissues assigned to two risk-groups of the PAC patients. Similarly to the results of the training dataset, the expression levels of LAMA3, E2F7, IFI44, and SLC12A2 were higher in PACs than in the adjacent tissues. However, LRIG1 mRNA was less expressed in PACs than in the adjacent tissues. In the analysis between the tumour tissues, mRNAs of LAMA3, E2F7, and IFI44 were more expressed in the tissues of the high-risk group than in those of low-risk group, whereas the expression levels of IFI44 and LRIG1 were lower in the high-risk group than in low-risk group (Figure S3A–E). Prognostic index values for the high-risk group were also significantly higher than those for the two other groups (Figure S3F).

Univariate Cox regression analysis revealed significant prognostic accuracy of PAC-5 signature for survival time in the training dataset [hazard ratio (HR) 1.781, 95% confidence interval (Cl) 1.105–2.873, *p* = 0.018]. Since the training dataset had no information for clinicopathological characteristics, the prognostic value of the PAC-5 signature could not be compared with prognostic covariates. Thus, to compare the prognostic value of our PAC-5 signature with prognostic covariates, univariate and multivariate Cox regression analyses were also performed using the combined validation datasets. In the univariate analysis, pathological grade, primary tumour size, lymph nodes metastasis, AJCC staging, and the PAC-5 signature were significantly associated with OS, compared to their referents, except for pathological grade 4. The significant covariates and our PAC-5 signature were used in multivariate analysis, in which pathological grade (G2 and G3), primary tumour size (T2 and T3), lymph nodes metastasis, and the PAC-5 signature still presented significant prognostic values (Table 1).

### 2.3. Validation of the PAC-5 Signature in Stage I and II PAC Patients

**Figure 4:**
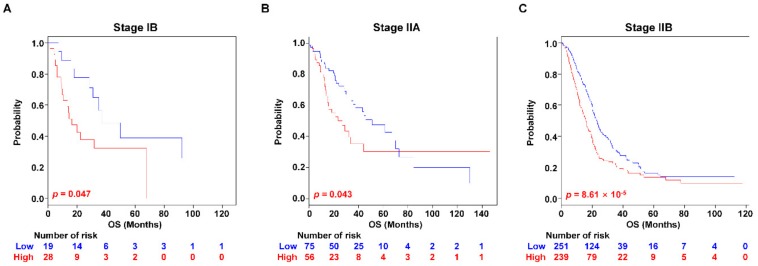
Kaplan–Meier survival analysis of PAC patients with stage IB, IIA, and IIB. (**A**–**C**) Kaplan–Meier survival analyses were performed to estimate the differences in OS between the low- and high-risk patients in stage IB, IIA and IIB. *p*-Values were computed by log-rank test.

The AJCC staging system is the most widely accepted prognostic model for PAC. However, the prognostic value of AJCC staging is indeed limited, by which the survival rates of patients in IB, IIA, and IIB are identical [3]. Thus, we investigated whether the PAC-5 signature could suitably stratify patients with stage I or II tumours into the two risk-groups in the validation datasets. The combined validation datasets included patients with survival information in stage I (*n* = 66, 9%) and II (*n* = 621, 85.4%). Indeed, we observed that the AJCC staging system has not properly stratified patients with stage IA, IB, IIA and IIB for survival (Figure S4A). Especially, Kaplan–Meier survival curves for the stage IB and IIA were not significantly different (*p* = 0.297, Figure S4B). However, the PAC-5 signature significantly stratified the stage IB or IIA patients into low- and high-risk groups (*p* = 0.047 for stage IB and *p* = 0.043 for stage IIA, respectively, Figure 4A,B). Moreover, it stratified the patients with stage IIB into two distinct prognostic risk-groups (*p* = 8.61 × 10^−5^, Figure 4C). The patients with stage IA could not be classified into different risk-groups by the PAC-5 signature (*p* = 0.109, Figure S4C).

### 2.4. Association of the PAC-5 Signature with Advantage of Adjuvant Therapies

#### 2.4.1. Chemotherapy

**Figure 5:**
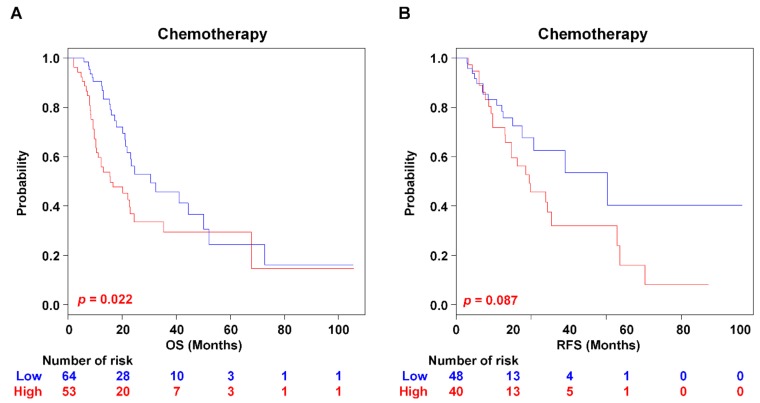
Kaplan–Meier survival analysis of PAC patients with chemotherapy. The patients were separated into risk-subgroups according to chemotherapy treatment. Kaplan–Meir analyses were used to evaluate the therapeutic advantage. (**A**) Kaplan–Meier plots for OS of two risk-subgroups. (**B**) Kaplan–Meier plots for RFS of two risk-subgroups. *p*-Values were computed by the log-rank test.

Gemcitabine-based adjuvant chemotherapy is currently recommended as a standard therapy after surgery for PAC [2]. However, the substantial number of PAC patients poorly respond to the chemotherapeutic agents, which rather causes drug-resistance and metastatic progression [6]. Actually, the clinicopathological information was incomplete for chemotherapeutic treatment in the GSE79668 and TCGA RNA-seq datasets (Table S5). The clinical information of TGCA dataset only indicated the patients who received the adjuvant chemotherapy (*n* = 100). In the case of GSE 79668 dataset, the details of chemotherapy information were archived with the drug names: Yes (*n* = 17) and No (*n* = 6). We could thus not assess the patients who had therapeutic benefit by the PAC-5 signature in each risk-group. However, the PAC-5 signature significantly stratified the patients who were chemotherapeutic drug-administered (*n* = 117) into two risk-subgroups for OS (*p* = 0.022, Figure 5A), but did not classify the patient for RFS (*p* = 0.087, Figure 5B).

#### 2.4.2. Radiotherapy

**Figure 6:**
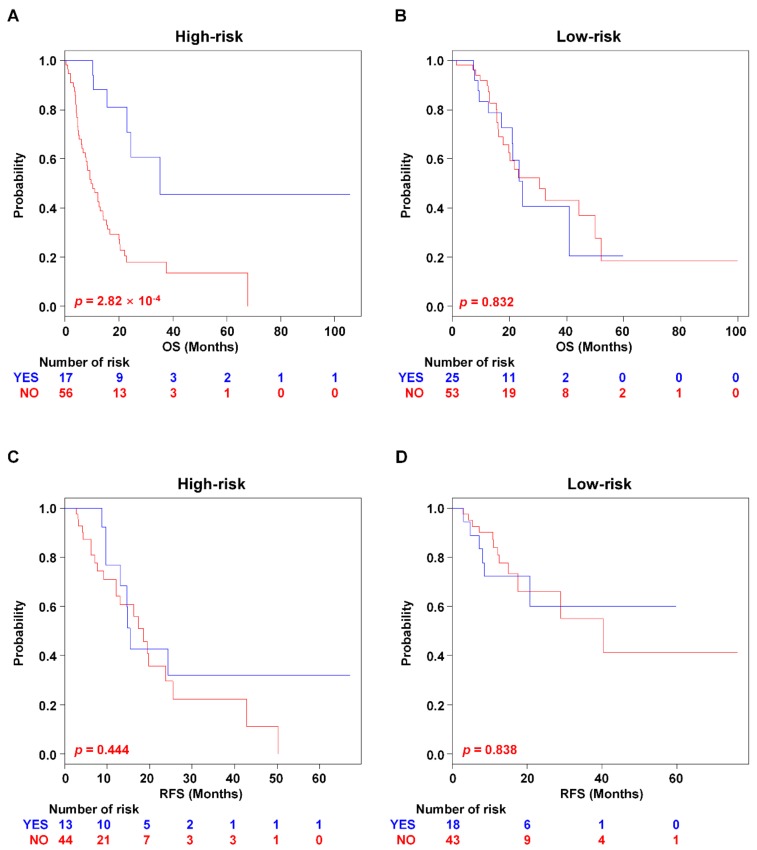
Kaplan–Meier survival analysis of PAC patients with radiation therapy. The patients were separated into risk-subgroups according to radiotherapy. Kaplan–Meir analyses were used to evaluate the therapeutic advantage. (**A**,**B**) Kaplan–Meier plots for OS of two risk-subgroups. (**C**,**D**) Kaplan–Meier plots for RFS of two risk-subgroups. *p*-Values were computed by the log-rank test.

Adjuvant radiotherapy has frequently been used as an integral component to treat PAC [17]. However, it is still controversial whether the patients benefit from radiotherapy [18]. Thus, in order to investigate the association of the PAC-5 signature with a response to adjuvant radiotherapy, we performed subgroup analysis. Radiotherapy itself showed therapeutic benefit for OS, but not for RFS in the GSE79668 and TCGA RNA-seq datasets (*p* = 4.81 × 10^−3^ for OS and *p* = 0.631 for RFS, Figure S5A,B, respectively). By incorporating the PAC-5 signature into radiotherapy information, the high-risk patients were shown to obtain the benefit for OS, compared to the patients without adjuvant radiotherapy (*p* = 2.82 × 10^−4^, Figure 6A). In contrast, low-risk patients did not show significant difference in radiotherapy effect (*p* = 0.832, Figure 6B). However, both low- and high-risk groups did not benefit from radiotherapy for RFS (Figure 6C,D).

#### 2.4.3. Targeted Molecular Therapy

**Figure 7:**
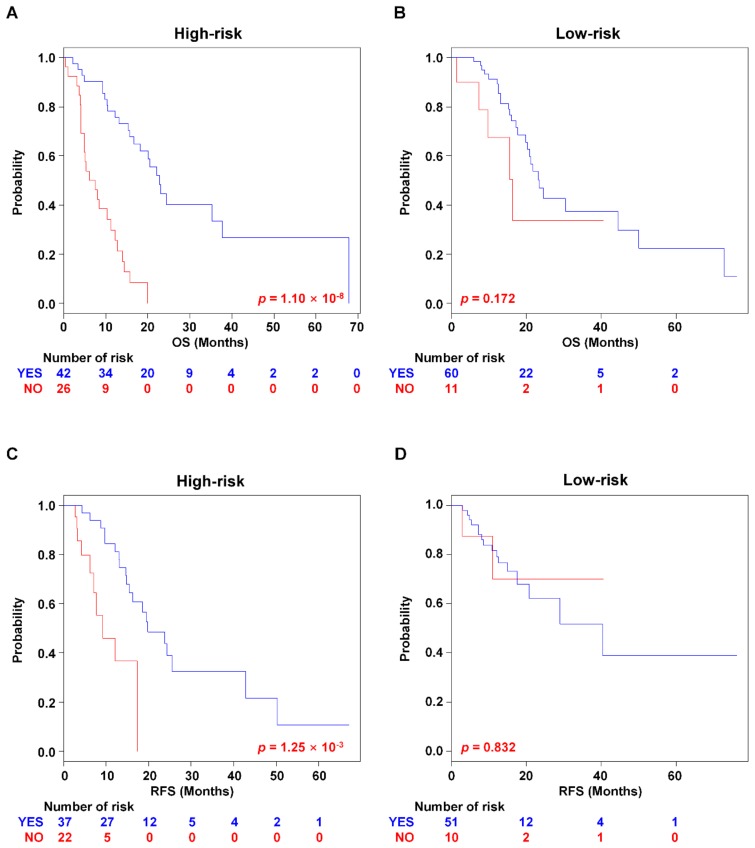
Kaplan–Meier survival analysis of PAC patients with targeted molecular therapy. The patients were separated into risk-subgroups according to targeted molecular therapy. Kaplan–Meir analyses were used to evaluate the therapeutic advantage. (**A**,**B**) Kaplan–Meier plots for OS of two risk-subgroups. (**C**,**D**) Kaplan–Meier plots for RFS of two risk-subgroups. *p*-Values were computed by the log-rank test.

Targeted molecular therapy has been suggested as a type of personalised medicine designed to treat cancer via inhibiting oncoproteins that drive signalling pathways in cancer [19]. In the PAC treatment, erlotinib, a selective epithermal growth factor receptor (EGFR) tyrosine kinase inhibitor (TKI), is the only targeted therapeutic agent approved by Food and Drug Administration (FDA) [20]. Although the administered drug was incompletely named in clinicopathological information of TCGA dataset, the association of the PAC-5 signature with the response to targeted molecular therapy was examined in the TCGA RNA-seq validation dataset. The patients with targeted molecular therapy had therapeutic benefit for OS, compared to the patients without targeted molecular therapy (*p* = 8.53 × 10^−4^ for OS and *p* = 0.018 for RFS, respectively, Figure S5C,D) By incorporating the PAC-5 signature into targeted molecular therapy, the high-risk patients were shown to obtain the benefit in OS and RFS compared to patients without targeted molecular therapy (*p* = 1.10 × 10^−8^ for OS and *p* = 1.25 ×10^−3^ for RFS, respectively, Figure 7A,C). In contrast, the low-risk patients did not show a significant difference in the treatment outcome (*p* = 0.172 for OS and *p* = 0.832 for RFS, respectively, Figure 7B,D).

### 2.5. Associations of PAC-5 Signature with KRAS Status

**Figure 8:**
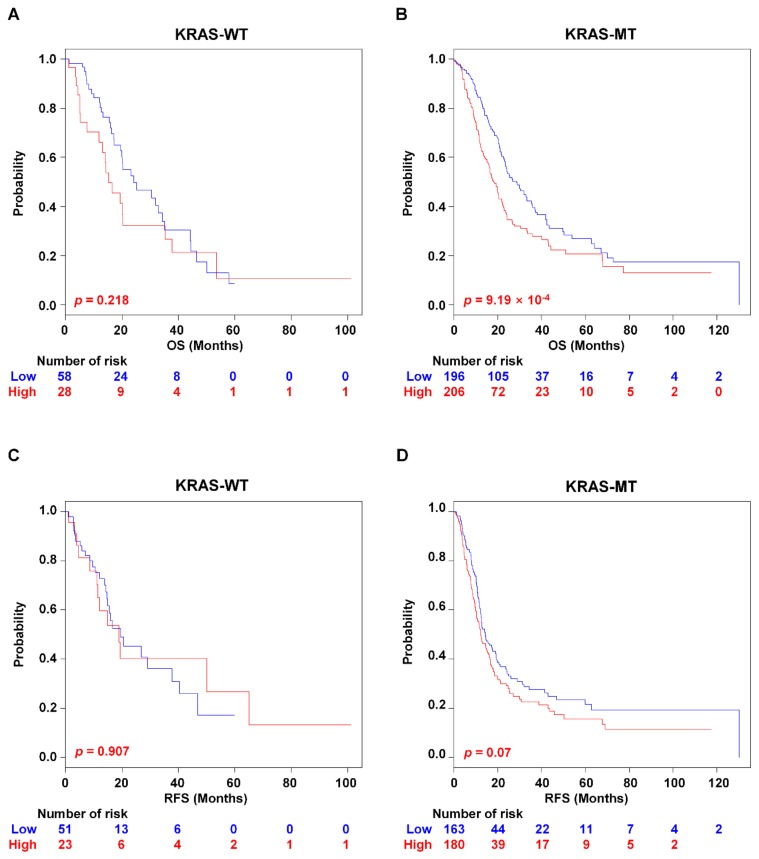
Kaplan–Meier survival analysis of PAC-5 gene signature with KRAS mutation. Kaplan–Meier survival analyses were used to estimate differences in OS and RFS between the low- and high-risk groups with KRAS status. (**A**,**B**) Kaplan–Meier plots for OS of two risk-subgroups. (**C**,**D**) Kaplan–Meier plots for RFS of two risk-subgroups. KRAS-WT, wild type KRAS; KRAS-MT, mutant KRAS. *p*-Values were computed by the log-rank test.

The malignant behaviour of cancer cells is compelled by mutations in oncogenes and tumour suppressor genes [21]. In PAC, *KRAS* is the most frequently mutated gene (in ~95% of cases) [22]. In the GSE79668 and TCGA RNA-seq datasets, *KRAS* mutations were observed in 82.4% of all pancreatic tumour cases. The *KRAS* status itself did not show significant difference in prognostic outcomes of the patients for both OS and RFS (Figure S6A,B). Although the PAC-5 signature did not classify the patients with *KRAS* wild type into low- and high-risk groups for OS (*p* = 0.218, Figure 8A), while the patients with *KRAS* mutants were significantly stratified into two distinct risk-groups (*p* = 9.19 × 10^−4^, Figure 8B). However, the PAC-5 signature did not still stratify the patients for RFS regardless of KRAS status (Figure 8C,D). To further assess whether the KRAS status influences the patients assigned to two risk-subgroups by the PAC-5 signature, we have stratified each risk-subgroup by incorporating the KRAS status. However, no risk-groups were further classified by KRAS status (Figure S7A–D).

### 2.6. DNA Methylation Regulating Expression of the PAC-5 Genes

**Figure 9:**
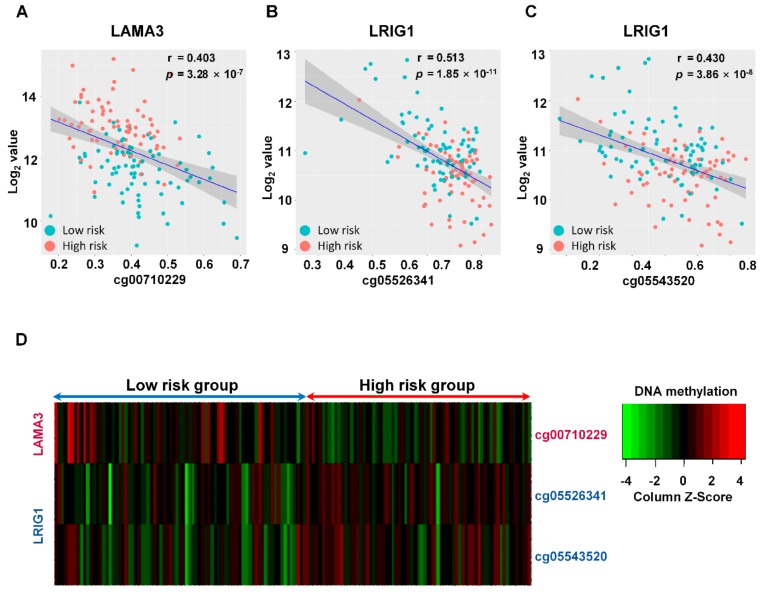
Methylation assessment of LAMA3 and LRIG1 genes in two risk-subgroups according to PAC-5 signature. Pearson’s correlation was used to measure linear relationships between DNA methylation and gene expression levels. *r*-Value indicated the Pearson’s correlation coefficient, and the *p*-Value (2-tailed) was the probability of a correlation. (**A**–**C**) Correlations between DNA methylations and the indicated genes. (**D**) Heatmap showed trend of the PAC-5 gene methylations according to the risk-subgroups. The colour keys of standardised methylation β values were presented in the legends, with red indicating hypermethylation and green indicating hypomethylation.

DNA methylation is a critical epigenetic gene regulation mechanism in cancer [23]. To assess whether the DNA methylation influenced the PAC-5 gene expression, correlations between the gene expression and their DNA methylation status at CpG sites were analysed. The threshold for the methylation value of CpG sites was set as the absolute value of Δβ = β_Tumour_ − β_Normal_ > 0.1 between the tumour and adjacent normal tissue; Δβ > 0.1 was defined as hypermethylated sites, and Δβ < –0.1 was considered as hypomethylated sites. The associations between the proximal gene expression and DNA methylation were determined by Pearson’s correlation coefficient (r). When the two criteria (*r* > 0.4 and *p* < 0.05) were satisfied, the correlation value was defined significant. The significant CpG sites were obtained for two genes, *LAMA3* and *LRIG1* with moderate *r*-Values (Figure 9A–C and Table S6). A DNA methylation heatmap for these three genes was provided in Figure 9D. However, no considerable CpG sites were found for regulation of the other genes.

### 2.7. Identification of Protein–Protein Interaction Network Associated with the PAC-5 Signature

**Figure 10:**
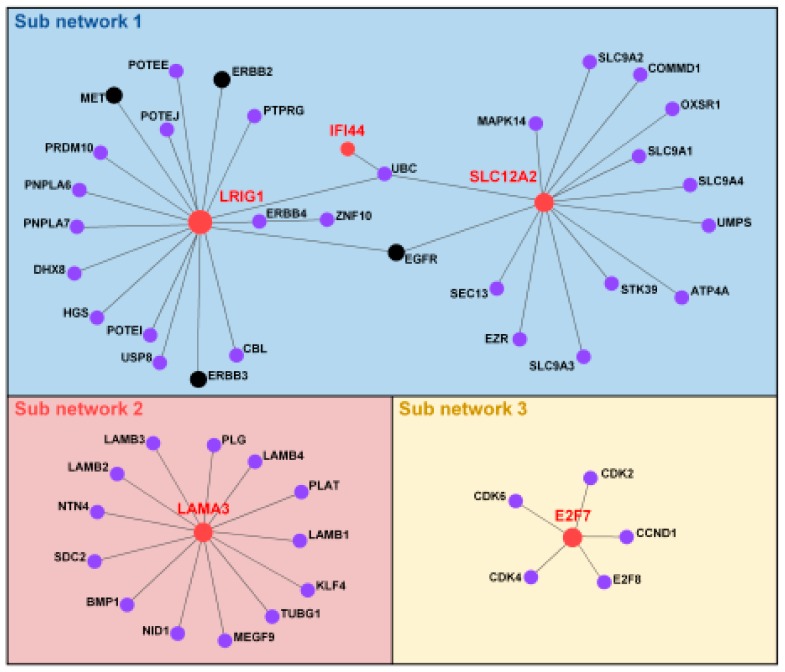
Protein–protein interaction network analysis of the PAC-5 genes. Interaction map was generated using the STRING database with experimental evidence in the Network Analyst 3.0. The proteins of the PAC-5 signature were red-circled, and the proteins related to the term of EGFR inhibitor resistance in KEGG pathway were black-circled.

Finally, to investigate how the PAC-5 genes might contribute to PAC progression, we employed PPI analysis in the NetworkAnalyst tool [24]. Three PPI networks related to the PAC-5 genes were generated with 53 nodes representing the proteins and 51 edges representing the interaction between the proteins (Figure 10). To further annotate functions of the proteins interacting with the PAC-5 genes, we executed a KEGG pathway analysis. Importantly, two genes in the PAC-5 signature, LRIG1 and SLC12A2 potentially interacted with the genes involved in EGFR-TKI resistance (Table S7).

## 3. Discussion

Pancreatic adenocarcinoma (PAC) is a highly heterogeneous disease with poor clinical outcomes. The prognostic prediction for treatment and mortality after surgery is frequently limited due to tumour molecular heterogeneity. Hence, the primary challenge is to develop a precise prognostic model that provides criteria for clinical treatment decisions. To address this issue, we established a PAC-5 gene expression signature via DEG profiling of normal pancreatic and PAC tissues in publicly available datasets to identify the tumour-specific genes. We here introduced novel genes, IFI44, SLC12A2 and LRIG1, which were not overlapped to other prognostic gene signatures for PAC. The robustness of the PAC-5 signature was supported by the reproducibility of a significant association between the predicted outcome and patient prognosis in external validation datasets composed with by far the largest gene expression profiles. Moreover, the PAC-5 signature could be a complementary prognostic adjunct to pathological staging to pave the way to personalised management strategies. Therapeutic subgroup analysis showed that PAC-5 signature might predict which patients would benefit from adjuvant therapies such as chemotherapy, radiotherapy, and targeted molecular therapy. Furthermore, we revealed that the PAC-5 signature could give potential therapeutic benefit to the patients with KRAS mutant. The five genes were found to be involved in tumourigenic signaling pathways, such as MAPK, PI3K-AKT, and ERBB pathways. Finally, network analyses of the PAC-5 signature provided clues for further elucidation of PAC heterogeneity and potential therapeutic target genes.

In the process of PAC-5 signature development, we initially subjected normal pancreas and tumour tissues to DEG analysis to find pancreatic cancer-specific genes. We subsequently identified seven genes (*LAMA3*, *E2F7*, *IFI44*, *SLC12A2*, *LRIG1*, *DUOXA1*, and *RBM1*) related to OS of PAC patients, using Cox proportional hazards analysis. Finally, we established a prognostic model with five genes that classified patients into two distinct risk-subgroups. Among the five genes in the PAC-5 signature, expression levels of *LAMA3*, *E2F7* and *IFI44* were elevated in the high-risk group, whereas *SLC12A12* and *LRIG1* expressions were relatively lowered. Interestingly, *SLC12A2* expression was higher in tumour tissues than in normal pancreas tissues; however, it was rather highly expressed in the low-risk group, compared to the high-risk one. Further observation is thus necessary to elucidate how *SLC12A2* expression is regulated in PAC biology. We also observed similar results from the analysis of an external validation dataset, which had adjacent tissues paired to their PAC tissues. A supervised method was used to construct the gene signature that was refined by LOOCV. Furthermore, a meta-analysis approach based on five microarray datasets (*n* = 474) and three RNA-seq datasets (*n* = 283) was applied to validate the prognostic significance of the gene signature in association with overall survival (30.4 months in the low-risk group and 17.7 months in the high-risk group). The PAC-5 signature and clinical parameter adjustment showed a significant association with survival in univariate analysis. Importantly, multivariate analysis demonstrated that the PAC-5 signature was the most significant variable associated with the prognosis of patients with PAC.

The AJCC staging system cannot accurately predict patient survival, by which the survival times of stage IB, IIA, and IIB patients actually show no significant differences [3]. This intra-stage variance is due to tumour heterogeneity, resulting in different clinical prognosis after receiving the same treatments [6]. In our subgroup analysis, the patients with stage IIB distinctively showed poor prognosis, not in accordance with previous studies. By incorporating the PAC-5 signature, the patients with stage IB, IIA, and IIB were further stratified into significantly low- and high-risk groups. These consistent results indicate that our gene signature could be a complementary prognostic adjunct to pathological staging to pave the way to personalised management strategies.

All current treatment regimens and many clinical trials targeting specific molecular pathways failed to improve therapeutic efficacy in PAC patients. Thus, the identification of patients who respond well to adjuvant therapy remains a major clinical concern. We demonstrated that the PAC-5 signature is closely associated with clinical outcomes of adjuvant therapies. Adjuvant chemotherapy is currently recommended as a standard therapy after resection for PAC [2]. However, it has modest clinical benefit and may not improve OS. The lack of significant chemotherapeutic response of PAC results in the inherent drug resistance of tumour cells [6]. In our analysis, the clinicopathological information was incomplete for chemotherapeutic treatment. We could thus not assess that the patients who had therapeutic benefit by the PAC-5 signature in each risk-group. Nonetheless, the PAC-5 signature further classified the patients who received the adjuvant chemotherapy for OS. The potential role of radiotherapy as management of resectable tumours in adjuvant settings remains controversial [17]. The treatment efficacy in many patients with PAC is conflicting [25]. In our study, subgroup analysis of patients with available data revealed that adjuvant radiotherapy was beneficial for high-risk patients to improve OS. Targeted molecular therapy is one of the primary modalities in cancer treatment, which interferes with specific molecules needed for tumourigenesis [26]. Currently, no effective targeted molecular therapies have been found for PAC. Because of a lack of information on adjuvant targeted molecular therapy, we could not comprehensively evaluate the efficacy of the PAC-5 signature to predict therapeutic outcomes. However, we found that the PAC-5 signature evidently improved OS and RFS in high-risk patients treated with targeted molecular therapy but not in the low-risk group. Hence, the PAC-5 signature results suggested a potential advantage of adjuvant therapies to patients in high-risk group, although we could not draw definite conclusions because of the small number of patients used in these analyses or incomplete information.

In most cases of PAC, oncogenic *KRAS* mutations, which initially drive pancreatic neoplasia, are prevalent [22]. With the substantial evidence that mutant KRAS is critical for PAC progression, it is extensively investigated as well [27]. However, no effective targeted therapies for KRAS have been established for PAC. In our analysis, the patients with KRAS status were not involved in prognostic outcomes. We found that the PAC-5 signature in combination with *KRAS* status further stratified patients for OS, while the signature did not show prognostic differences for RFS. Thus, the PAC-5 signature might give the potential benefit from adjuvant TMT in patients with KRAS mutant type, although we agree that it would not be enough to make a strong conclusion for the predictive power due to the small number of patients used in these analyses.

The majority of genes in the PAC-5 signature (*LAMA3*, *E2F7*, *SLC12A2*, and *LRIG1*) have been reported to be associated with tumour progression in various types of cancer. LAMA3 is the alpha subunit of laminin-332, which is further composed of laminin subunit β2 (LAMB2) and laminin subunit γ2 (LAMC2). Tumourigenic roles of the laminin-332 are well-known in diverse cancers such as breast and colon cancers [28] and squamous cell carcinoma [29] as well as PAC [30]. E2F7, one of the E2F transcription factors, has critical roles in the regulation of cell cycle progression and DNA-damage response [31,32]. In cancer biology, E2F7 is associated with poor survival in squamous cancers [33]. Loss of E2F7 confers resistance to poly-ADP-ribose polymerase (PARP) inhibitors in BRCA2-deficient breast cancer cells [34]. SLC12A2 plays a role in Na^+^, K^+^, and 2Cl-cotransporter in membrane blebbing via interactions with actin and the p38 mitogen-activated protein kinases (*p*38 MAPK) in malignant mesothelioma cells [35]. Pharmacological modulation of K^+^ transport increases sensitivity to apoptosis in human malignant pleural mesothelioma cell line [36]. SLC12A2 expression is associated with glioblastoma cell invasion and aggressiveness [37]. LRIG1 participates in the aggressive progression of several tumours, in which its expression is frequently decreased [38,39,40]. More importantly, it blocks the EGFR pathway with its antagonist erlotinib abrogated LRIG1 suppression-induced EMT and, subsequently, cell invasion, migration, and vasculogenic mimicry of melanoma cells under hypoxia [41]. *IFI44* is one of the interferon-α stimulated genes (ISGs) which is associated with infections of several viruses such as hepatitis C virus [42], rhinovirus [43] and human papillomavirus [44]. In addition, IFI44 inhibits cAMP-mediated signalling downstream of ERK via depletion of intracellular GTP, resulting in arrest of cell division in melanoma [45]. In breast cancer, reduced expression of IFI44 in lymphocytes exacerbates cancer-associated immune dysfunction [46]. However, the molecular functions of IFI44 in cancer cells remain to be explored.

The PPI network analysis of the PAC-5 signature indicated the possibility of drug resistance to EGFR-TKIs. EGFR-TKIs generally bind the tyrosine kinase domain of EGFR, and thus inhibit its activity. For instance, erlotinib and/or gefitinib (small molecular EGFR-TKIs) achieved significant treatment efficacy in patients with lung cancer or PAC [20,47]. Nevertheless, cancer cells gradually acquire resistance to these drugs, resulting in progression and relapse [48]. SLC12A2 and LRIG1 were shown to interact with proteins involved in EGFR kinase inhibitor resistance, such as EGFR, ERBB2, ERBB3, and c-MET. ERBBs are known to promote pancreatic cancer development [49]. Overexpression or mutation of ERBB2 is associated with resistance to EGFR-TKIs [50]. Overexpression of ERBB3 in poorly differentiated colorectal cancer cell lines led to a significant resistance to gefitinib in vitro and in vivo [51]. Furthermore, ERBB3 phosphorylation is driven by EGFR and/or ERBB2, or through amplification of the proto-oncogene c-Met [52]. Several studies have shown that the drug resistance to either TKIs or gemcitabine is developed through hyperactivation of the c-MET/HGF signalling axis [53,54,55]. In addition, although LAMA3 and E2F7 did not exhibit direct interactions with proteins involved in resistance to EGFR-TKIs, these two proteins were also connected to many proteins related to tumour progression [56,57]. Accordingly, we suggest that the PAC-5 signature can be used as a biomarker panel to estimate not only the clinical effectiveness of EGFR-TKIs but also drug resistance. In this manner, the PAC-5 genes might be utilised as valuable targets for concurrent therapy in addition to their role as prognostic markers.

The development of high-throughput technologies has allowed accessing integrated approaches of the genetic and epigenetic patterns for the regulatory mechanism of interest genes. In our analysis of DNA methylation for gene regulations, we found that the genomic alterations in methylation influence the PAC-5 gene expressions. DNA methylation is critical in the early formation and process of diseases, especially for cancers, and the hypermethylation of promotor or/and CpG island (CGI) of genes results in the transcriptional silencing [58]. The *LAMA3* loci were relatively hypermethylated at one transcriptional region in the patients of the low-risk group, which were significantly associated with the mRNA expressions. In coincidence with our data, a previous study reports that *LAMA3* promoter methylation frequency was inversely associated with increased tumour stage and tumour size in breast cancer [59]. In contrast, two different CpG regions of *LRIG1* were hypermethylated in patients with high-risk, which were significantly involved in the gene expression. At present, additional regulatory mechanisms for the five gene expressions in PAC biology remain to be uncovered. Perspective studies would provide new insight on cancer-specific gene regulations for understanding the molecular heterogeneity of PAC.

## 4. Methods 

### 4.1. PAC Patient and Gene Expression Data

All clinical and gene expression data for PAC patients were obtained from the Gene Expression Omnibus database (http://www.ncbi.nlm.nih.gov/geo/), ArrayExpress (http://www.ebi.ac.uk/arrayexpress/), International Cancer Genome Consortium (ICGC, http://icgc.org) and The Cancer Genome Atlas (TCGA, http://www.cancer.gov/about-nci/organization/ccg/research/structural-genomics/tcga/). All of the used datasets contained clinical information of patients on survival event and time. In the case of the TCGA dataset, the gene expression data, methylation and clinical information were obtained from the University of California Santa Cruz (http://xena.ucsc.edu/). Tumour tissues in two RNA-seq datasets of TCGA and ICGC contained pancreatic neuroendocrine tumours and other types of carcinoma. To discriminate the PAC tissues, the histological subtypes provided in clinical information of TCGA and ICGC were reviewed according to guidelines [18,60]. The gene expression data were normalised using a robust multiarray averaging method (RMA) [61]. The 636 patients of five microarray and three RNA sequencing datasets were used in the analysis. GSE71729 (Agilent-014850 Whole Human Genome Microarray 4x44K G4112F) [13] dataset had gene expression data of patients from multiple cancers, in which 125 PAC patients and 46 normal pancreas subjects were included. The GSE71729 was used as the training dataset to establish a gene signature. The validation datasets were GSE17891 (*n* = 27, Affymetrix Human Genome U133 Plus 2.0 Array) [16], GSE57495 (*n* = 63, Rosetta/Merck Human RSTA Custom Affymetrix 2.0 microarray) [62], GSE62452 (*n* = 66, Affymetrix Human Gene 1.0 ST Array) [63], E-MEXP-2780 (*n* = 30, Affymetrix Human Genome U133 Plus 2.0) [64], E-MTAB-6134 (*n* = 288, Affymetrix Human Genome U219 Array) [60], GSE79668 (*n* = 51) [65], ICGC (*n* = 82) [14] and TCGA (*n* = 150) [15]. In the validation datasets, the majority of patients were assigned to stage IIB disease (490/727, 64.7%). Notably, 117/201 patients received chemotherapy (58.2%), 42/187 patients received radiotherapy (22.5%), and 102/139 patients received targeted molecular therapy (73.4%). A summary of the training and validation datasets is provided in Table S1.

### 4.2. Development of the Prognostic Gene Expression Signature

A prognostic gene signature was developed using the GSE71729 training dataset. First, the 19,749 genes were filtered by at least more than two folds of the absolute value of log_2_ scale in less than 20% of the patients. Next, differentially expressed genes (DEGs) analysis [66] between normal pancreas (*n* = 46) and PAC tissues (*n* = 125) was performed to isolate tumour-specific genes. Stringent *p*-Value (*p* < 0.001) and false discovery rate (FDR) < 0.1 using univariate permutation test (1000 times) were set as the cutoffs for the DEGs. The LASSO regression [67] (*p* < 0.001) was then used to identify the OS-associated gene signature from the training dataset. After this step, genes that were not compatible with the external validation datasets or not significant in the individual survival curves were excluded. For predicting prognosis, genes from the survival signature were applied to survival risk prediction analysis. This method utilised the principal component from the training dataset and generated a prognostic index for each patient. The prognostic index (*y*) was computed by the formula where *wi* and *xi* were the weight and logged gene expression for the *i*-th gene, respectively, as below.

$$y=\overset{i}{\underset{n=1}{\sum }}\left(iwi\cdot xi\right)-0.037658$$

The weight values of all genes were as follows: *w_LAMA3_*, 0.306929; *w_E2F7_*, 0.118701; *w_IFI44_*, 0.263742; *w_SLC12A2_*, −0.4137 and *w_LRIG1_*, −0.190012. The patients were divided into two risk-groups according to a median prognostic index value of −0.060239. Patients were assigned to the high-risk group if their prognostic index values were higher than the median value, whereas the low-risk group comprised patients with the prognostic index values that were equivalent to or less than the median value. Dendrogram of prognostic genes was generated using the heatmap function in R, using default settings for the clustering algorithm.

### 4.3. Validation of the Prognostic Signature

The validation of the gene signature was performed in external datasets. Gene expression data from different datasets were normalised by subtracting the median expression value across the samples. Compound covariate predictor was utilised as a class prediction algorithm to further refine this model and sub-stratify the predicted outcomes [68]. The robustness was estimated by the misclassification rate that was determined during leave-one-out cross-validation (LOOCV).

Kaplan–Meier survival analyses were performed after the patient classification into two risk-groups, and Chi-square (χ^2^) and log-rank tests were used to evaluate the survival probability in the two predicted risk-subgroups of patients. Univariate and multivariate Cox proportional hazard regression analyses were used to evaluate independent prognostic factors associated with survival, and the gene signature, tumour grade, and pathological characteristics were employed as covariates. 

### 4.4. Network and Pathway Enrichment Analysis

NetworkAnalyst 3.0 is a web-based visual analytics platform for comprehensive profiling, meta-analysis and systems-level interpretation of gene expression data (http://www.networkanalyst.ca/) [24], accessed July 2019. The NetworkAnalyst 3.0 was used to generate protein–protein interaction (PPI) networks, and then to perform KEGG pathway enrichment analysis. The PPI network analysis was performed using STRING database v11.0 (http://string-db.org/) [69] with experimental evidence. KEGG pathway enrichment analysis was conducted to annotate the pathways, in which the genes in expression signature were involved. An adjusted *p* < 0.05 was considered significant for all enrichment analyses.

### 4.5. DNA Methylation Analysis of Gene Regulation

DNA methylation profiling analysis for the gene regulations was performed using TCGA DNA methylation data (Illumina Infinium HumanMethylation450 platform). The DNA methylation β values for CpG sites indicated the estimate of methylation level using the ratio of intensities between methylated and unmethylated alleles. The threshold for the methylation value of a CpG site was set as the absolute value of Δβ = β_Tumour_ − β_Normal_ > 0.1 between the tumour and adjacent normal tissue; Δβ > 0.1 was defined as a hypermethylation, while Δβ < −;0.1 was determined as a hypomethylation. The association between the proximal gene expression and DNA methylation was measured by Pearson’s correlation coefficient (r). The correlation values were indicated: 0.1 < |r| ≤ 0.4, weak correlation; 0.4 < |r| ≤ 0.7, moderate correlation; r = 0.7 < |r| ≤ 0.9, strong correlation.

### 4.6. Statistical Methods

Gene expression datasets were analysed using BRB-Array Tools Version 4.6 (http://brb.nci.nih.gov/BRB-ArrayTools/) [66]. All other statistical analyses were accomplished in the R language environment (http://www.r-project.org) and Statistical Package for Social Sciences (SPSS) software (version 25, SPSS Inc, Chicago, IL, USA). In all statistical analyses, a *p*-Value of less than 0.05 was considered significant.

## 5. Conclusions

In this study, we developed a novel gene signature, the PAC-5 signature, which was developed via DEG between the normal pancreas and PAC tissues to identify the cancer-specific genes. The gene signature accurately and robustly predicts individual PAC patients at high risk of mortality. The prognostic value of the PAC-5 signature was statistically significant in the overall datasets, independently of the pathological staging. Furthermore, we provided evidence that the PAC-5 signature might help to refine the selection of the PAC patients who are beneficial from adjuvant radiation or targeted molecular therapies. Hence, we propose that the five genes in our signature might be promising molecular targets for PAC treatment.

## Figures and Tables

**Table 1:** Univariate and multivariate Cox proportional hazard regression analyses of clinical variable in validation datasets.

Variables		OS					
		Univariate			Multivariate		
		HR	95% CI	*p*-Value	HR	95% CI	*p*-Value
**Age**							
	**≤65 (referent)**	1					
	**>65**	1.316	0.997–1.738	0.053			
**Gender**							
	**Female (referent)**	1					
	**Male**	0.898	0.735–1.097	0.292			
**Family history**							
	**No (referent)**	1					
	**Yes**	0.862	0.476–1.560	0.624			
**Race**							
	**Black (referent)**	1					
	**Other**	1.246	0.544–2.856	0.614			
**Pancreatitis**							
	**No (referent)**	1					
	**Yes**	1.033	0.491–2.173	0.931			
**Diabetes**							
	**No (referent)**	1					
	**Yes**	0.817	0.542–1.230	0.332			
**Grade**							
	**G1 (referent)**	1			1		
	**G2**	1.363	1.047–1.773	0.021	1.324	1.005–1.744	0.046
	**G3**	2.270	1.706–3.022	1.89 × 10^−8^	1.924	1.410–2.627	3.72 × 10^−5^
	**G4**	1.634	0.515–5.182	0.405	2.399	0.746–7.718	0.142
**T**							
	**T1 (referent)**	1			1		
	**T2**	2.010	1.018–3.970	0.044	2.309	1.015–5.524	0.046
	**T3**	2.434	1.295–4.574	0.006	2.778	1.199–6.438	0.017
	**T4**	2.966	1.168–7.534	0.022	5.418	0.643–45.632	0.120
**N**							
	**N0 (referent)**	1			1		
	**N1**	1.938	1.504–2.496	3.08 × 10^−7^	2.021	1.466–2.787	1.76 × 10^−5^
**AJCC Staging**							
	**1 (referent)**	1			1		
	**2**	1.611	1.140–2.277	0.007	0.676	0.342–1.338	0.261
	**3**	2.173	1.215–3.888	0.009	0.420	0.025–7.122	0.548
	**4**	2.735	1.346–5.555	0.005	0.590	0.130–2.675	0.494
**PAC-5**							
	**Low (referent)**	1			1		
	**High**	1.599	1.333–1.895	2.41 × 10^−7^	1.349	1.080–1.685	0.008

AJCC, American Joint Committee on Cancer; OS, overall survival; HR, hazard ratio; CI, Confidence Interval; T, primary tumour size; N, lymph node metastasis. The Wald test was used to estimate *p*-Values. All statistical tests were two-sided.

## Supplementary Materials

The following are available online at https://www.mdpi.com/2072-6694/11/11/1749/s1. Figure S1: Comparison of PAC-5 gene patterns between normal pancreas tissues and tumour tissues assigned to low- or high-risk groups in the training dataset, Figure S2: Kaplan–Meier survival analysis of PAC-5 signature in microarray and RNA-seq datasets, Figure S3: Comparison of PAC-5 gene patterns between normal pancreas tissues and tumour tissues assigned to low- or high-risk groups in the GSE62452 validation dataset, Figure S4: Kaplan–Meier survival analysis of PAC patients with early-stage, Figure S5: Kaplan–Meier survival analysis according to radiotherapy and targeted molecular therapy, Figure S6: Kaplan–Meier survival analysis of PAC patients with KRAS status, Figure S7: Kaplan–Meier survival analysis of PAC-5 risk-subgroups with KRAS status, Table S1: Clinical characteristics of patients in training and validation datasets, Table S2: Class comparison analysis between normal pancreas and pancreatic tumour tissues, Table S3: Annotation of the PAC-5 genes, Table S4: Association between the PAC-5 signature and Moffitt classification, Table S5: Information of chemotherapeutic drug names and treatment in validation datasets, Table S6: Methylation of the PAC-5 genes, Table S7: List of protein–protein interaction network.

## References

[1] Siegel R.L., Miller K.D., Jemal A. (2019). Cancer statistics, 2019. CA Cancer J. Clin..

[2] Kindler H.L. (2018). A Glimmer of Hope for Pancreatic Cancer. N. Engl. J. Med..

[3] van Roessel S., Kasumova G.G., Verheij J., Najarian R.M., Maggino L., de Pastena M., Malleo G., Marchegiani G., Salvia R., Ng S.C. (2018). International Validation of the Eighth Edition of the American Joint Committee on Cancer (AJCC) TNM Staging System in Patients With Resected Pancreatic Cancer. JAMA Surg..

[4] Conroy T., Desseigne F., Ychou M., Bouche O., Guimbaud R., Becouarn Y., Adenis A., Raoul J.L., Gourgou-Bourgade S., de la Fouchardiere C. (2011). FOLFIRINOX versus gemcitabine for metastatic pancreatic cancer. N. Engl. J. Med..

[5] Von Hoff D.D., Ervin T., Arena F.P., Chiorean E.G., Infante J., Moore M., Seay T., Tjulandin S.A., Ma W.W., Saleh M.N. (2013). Increased survival in pancreatic cancer with nab-paclitaxel plus gemcitabine. N. Engl. J. Med..

[6] Juiz N.A., Iovanna J., Dusetti N. (2019). Pancreatic Cancer Heterogeneity Can Be Explained Beyond the Genome. Front. Oncol..

[7] Gerlinger M., Rowan A.J., Horswell S., Math M., Larkin J., Endesfelder D., Gronroos E., Martinez P., Matthews N., Stewart A. (2012). Intratumor heterogeneity and branched evolution revealed by multiregion sequencing. N. Engl. J. Med..

[8] Karamitopoulou E. (2019). Tumour microenvironment of pancreatic cancer: Immune landscape is dictated by molecular and histopathological features. Br. J. Cancer.

[9] Neesse A., Bauer C.A., Ohlund D., Lauth M., Buchholz M., Michl P., Tuveson D.A., Gress T.M. (2019). Stromal biology and therapy in pancreatic cancer: Ready for clinical translation?. Gut.

[10] Toustrup K., Sorensen B.S., Nordsmark M., Busk M., Wiuf C., Alsner J., Overgaard J. (2011). Development of a hypoxia gene expression classifier with predictive impact for hypoxic modification of radiotherapy in head and neck cancer. Cancer Res..

[11] Cardoso F., van’t Veer L.J., Bogaerts J., Slaets L., Viale G., Delaloge S., Pierga J.Y., Brain E., Causeret S., DeLorenzi M. (2016). 70-Gene Signature as an Aid to Treatment Decisions in Early-Stage Breast Cancer. N. Engl. J. Med..

[12] Haider S., Wang J., Nagano A., Desai A., Arumugam P., Dumartin L., Fitzgibbon J., Hagemann T., Marshall J.F., Kocher H.M. (2014). A multi-gene signature predicts outcome in patients with pancreatic ductal adenocarcinoma. Genome Med..

[13] Moffitt R.A., Marayati R., Flate E.L., Volmar K.E., Loeza S.G., Hoadley K.A., Rashid N.U., Williams L.A., Eaton S.C., Chung A.H. (2015). Virtual microdissection identifies distinct tumor- and stroma-specific subtypes of pancreatic ductal adenocarcinoma. Nat. Genet..

[14] Bailey P., Chang D.K., Nones K., Johns A.L., Patch A.M., Gingras M.C., Miller D.K., Christ A.N., Bruxner T.J., Quinn M.C. (2016). Genomic analyses identify molecular subtypes of pancreatic cancer. Nature.

[15] (2017). The Cancer Genome Atlas Research Network, Integrated Genomic Characterization of Pancreatic Ductal Adenocarcinoma. Cancer Cell.

[16] Collisson E.A., Sadanandam A., Olson P., Gibb W.J., Truitt M., Gu S., Cooc J., Weinkle J., Kim G.E., Jakkula L. (2011). Subtypes of pancreatic ductal adenocarcinoma and their differing responses to therapy. Nat. Med..

[17] Kooby D.A., Gillespie T.W., Liu Y., Byrd-Sellers J., Landry J., Bian J., Lipscomb J. (2013). Impact of adjuvant radiotherapy on survival after pancreatic cancer resection: An appraisal of data from the national cancer data base. Ann. Surg. Oncol..

[18] Gill A.J., Klimstra D.S., Lam A.K., Washington M.K. (2019). Tumours of the Pancreas. WHO Classification of Tumours of the Digestive System.

[19] Vander Heiden M.G. (2011). Targeting cancer metabolism: A therapeutic window opens. Nat. Rev. Drug Discov..

[20] Moore M.J., Goldstein D., Hamm J., Figer A., Hecht J.R., Gallinger S., Au H.J., Murawa P., Walde D., Wolff R.A. (2007). Erlotinib plus gemcitabine compared with gemcitabine alone in patients with advanced pancreatic cancer: A phase III trial of the National Cancer Institute of Canada Clinical Trials Group. J. Clin. Oncol..

[21] Hanahan D., Weinberg R.A. (2011). Hallmarks of cancer: The next generation. Cell.

[22] Bryant K.L., Mancias J.D., Kimmelman A.C., Der C.J. (2014). KRAS: Feeding pancreatic cancer proliferation. Trends Biochem. Sci..

[23] Jones P.A., Issa J.P., Baylin S. (2016). Targeting the cancer epigenome for therapy. Nat. Rev. Genet..

[24] Zhou G., Soufan O., Ewald J., Hancock R.E.W., Basu N., Xia J. (2019). NetworkAnalyst 3.0: A visual analytics platform for comprehensive gene expression profiling and meta-analysis. Nucleic Acids Res..

[25] Chargari C., Soria J.C., Deutsch E. (2013). Controversies and challenges regarding the impact of radiation therapy on survival. Ann. Oncol..

[26] Schilsky R.L. (2014). Implementing personalized cancer care. Nat. Rev. Clin. Oncol..

[27] Waters A.M., Der C.J. (2018). KRAS: The Critical Driver and Therapeutic Target for Pancreatic Cancer. Cold Spring Harb. Perspect Med..

[28] Koshikawa N., Giannelli G., Cirulli V., Miyazaki K., Quaranta V. (2000). Role of cell surface metalloprotease MT1-MMP in epithelial cell migration over laminin-5. J. Cell Biol..

[29] Tran M., Rousselle P., Nokelainen P., Tallapragada S., Nguyen N.T., Fincher E.F., Marinkovich M.P. (2008). Targeting a tumor-specific laminin domain critical for human carcinogenesis. Cancer Res..

[30] Fukushima N., Sakamoto M., Hirohashi S. (2001). Expression of laminin-5-gamma-2 chain in intraductal papillary-mucinous and invasive ductal tumors of the pancreas. Mod. Pathol..

[31] Chen H.Z., Tsai S.Y., Leone G. (2009). Emerging roles of E2Fs in cancer: An exit from cell cycle control. Nat. Rev. Cancer.

[32] Carvajal L.A., Hamard P.J., Tonnessen C., Manfredi J.J. (2012). E2F7, a novel target, is up-regulated by p53 and mediates DNA damage-dependent transcriptional repression. Genes Dev..

[33] Hazar-Rethinam M., de Long L.M., Gannon O.M., Boros S., Vargas A.C., Dzienis M., Mukhopadhyay P., Saenz-Ponce N., Dantzic D.D., Simpson F. (2015). RacGAP1 Is a Novel Downstream Effector of E2F7-Dependent Resistance to Doxorubicin and Is Prognostic for Overall Survival in Squamous Cell Carcinoma. Mol. Cancer Ther..

[34] Clements K.E., Thakar T., Nicolae C.M., Liang X., Wang H.G., Moldovan G.L. (2018). Loss of E2F7 confers resistance to poly-ADP-ribose polymerase (PARP) inhibitors in BRCA2-deficient cells. Nucleic Acids Res..

[35] Janson V., Andersson B., Behnam-Motlagh P., Engstrom K.G., Henriksson R., Grankvist K. (2008). Acquisition of cisplatin-resistance in malignant mesothelioma cells abrogates Na+, K+, 2Cl(-)-cotransport activity and cisplatin-induced early membrane blebbing. Cell Physiol. Biochem..

[36] Andersson B., Behnam-Motlagh P., Henriksson R., Grankvist K. (2005). Pharmacological modulation of lung cancer cells for potassium ion depletion. Anticancer Res..

[37] Garzon-Muvdi T., Schiapparelli P., ap Rhys C., Guerrero-Cazares H., Smith C., Kim D.H., Kone L., Farber H., Lee D.Y., An S.S. (2012). Regulation of brain tumor dispersal by NKCC1 through a novel role in focal adhesion regulation. PLoS Biol..

[38] Chang L., Shi R., Yang T., Li F., Li G., Guo Y., Lang B., Yang W., Zhuang Q., Xu H. (2013). Restoration of LRIG1 suppresses bladder cancer cell growth by directly targeting EGFR activity. J. Exp. Clin. Cancer Res..

[39] Tanemura A., Nagasawa T., Inui S., Itami S. (2005). LRIG-1 provides a novel prognostic predictor in squamous cell carcinoma of the skin: Immunohistochemical analysis for 38 cases. Dermatol. Surg..

[40] Thompson P.A., Ljuslinder I., Tsavachidis S., Brewster A., Sahin A., Hedman H., Henriksson R., Bondy M.L., Melin B.S. (2014). Loss of LRIG1 locus increases risk of early and late relapse of stage I/II breast cancer. Cancer Res..

[41] Li W., Zhou Y. (2019). LRIG1 acts as a critical regulator of melanoma cell invasion, migration, and vasculogenic mimicry upon hypoxia by regulating EGFR/ERK-triggered epithelial-mesenchymal transition. Biosci. Rep..

[42] Kitamura A., Takahashi K., Okajima A., Kitamura N. (1994). Induction of the human gene for p44, a hepatitis-C-associated microtubular aggregate protein, by interferon-alpha/beta. Eur. J. Biochem..

[43] Bochkov Y.A., Hanson K.M., Keles S., Brockman-Schneider R.A., Jarjour N.N., Gern J.E. (2010). Rhinovirus-induced modulation of gene expression in bronchial epithelial cells from subjects with asthma. Mucosal. Immunol..

[44] Kaczkowski B., Rossing M., Andersen D.K., Dreher A., Morevati M., Visser M.A., Winther O., Nielsen F.C., Norrild B. (2012). Integrative analyses reveal novel strategies in HPV11, -16 and -45 early infection. Sci. Rep..

[45] Hallen L.C., Burki Y., Ebeling M., Broger C., Siegrist F., Oroszlan-Szovik K., Bohrmann B., Certa U., Foser S. (2007). Antiproliferative activity of the human IFN-alpha-inducible protein IFI44. J. Interferon Cytokine Res..

[46] Critchley-Thorne R.J., Simons D.L., Yan N., Miyahira A.K., Dirbas F.M., Johnson D.L., Swetter S.M., Carlson R.W., Fisher G.A., Koong A. (2009). Impaired interferon signaling is a common immune defect in human cancer. Proc. Natl. Acad. Sci. USA.

[47] Mu X.L., Li L.Y., Zhang X.T., Wang M.Z., Feng R.E., Cui Q.C., Zhou H.S., Guo B.Q. (2005). Gefitinib-sensitive mutations of the epidermal growth factor receptor tyrosine kinase domain in chinese patients with non-small cell lung cancer. Clin. Cancer Res..

[48] Huang L., Fu L. (2015). Mechanisms of resistance to EGFR tyrosine kinase inhibitors. Acta Pharm. Sin. B.

[49] Oliveira-Cunha M., Newman W.G., Siriwardena A.K. (2011). Epidermal growth factor receptor in pancreatic cancer. Cancers.

[50] Landi L., Cappuzzo F. (2013). HER2 and lung cancer. Expert Rev. Anticancer Ther..

[51] Nakata S., Tanaka H., Ito Y., Hara M., Fujita M., Kondo E., Kanemitsu Y., Yatabe Y., Nakanishi H. (2014). Deficient HER3 expression in poorly-differentiated colorectal cancer cells enhances gefitinib sensitivity. Int. J. Oncol..

[52] Engelman J.A., Zejnullahu K., Mitsudomi T., Song Y., Hyland C., Park J.O., Lindeman N., Gale C.M., Zhao X., Christensen J. (2007). MET amplification leads to gefitinib resistance in lung cancer by activating ERBB3 signaling. Science.

[53] Ozasa H., Oguri T., Maeno K., Takakuwa O., Kunii E., Yagi Y., Uemura T., Kasai D., Miyazaki M., Niimi A. (2014). Significance of c-MET overexpression in cytotoxic anticancer drug-resistant small-cell lung cancer cells. Cancer Sci..

[54] Hage C., Rausch V., Giese N., Giese T., Schonsiegel F., Labsch S., Nwaeburu C., Mattern J., Gladkich J., Herr I. (2013). The novel c-Met inhibitor cabozantinib overcomes gemcitabine resistance and stem cell signaling in pancreatic cancer. Cell Death Dis..

[55] Moschetta M., Basile A., Ferrucci A., Frassanito M.A., Rao L., Ria R., Solimando A.G., Giuliani N., Boccarelli A., Fumarola F. (2013). Novel targeting of phospho-cMET overcomes drug resistance and induces antitumor activity in multiple myeloma. Clin. Cancer Res..

[56] Marinkovich M.P. (2007). Tumour microenvironment: Laminin 332 in squamous-cell carcinoma. Nat. Rev. Cancer.

[57] Endo-Munoz L., Dahler A., Teakle N., Rickwood D., Hazar-Rethinam M., Abdul-Jabbar I., Sommerville S., Dickinson I., Kaur P., Paquet-Fifield S. (2009). E2F7 can regulate proliferation, differentiation, and apoptotic responses in human keratinocytes: Implications for cutaneous squamous cell carcinoma formation. Cancer Res..

[58] Heyn H., Esteller M. (2012). DNA methylation profiling in the clinic: Applications and challenges. Nat. Rev. Genet..

[59] Sathyanarayana U.G., Padar A., Huang C.X., Suzuki M., Shigematsu H., Bekele B.N., Gazdar A.F. (2003). Aberrant promoter methylation and silencing of laminin-5-encoding genes in breast carcinoma. Clin. Cancer Res..

[60] Puleo F., Nicolle R., Blum Y., Cros J., Marisa L., Demetter P., Quertinmont E., Svrcek M., Elarouci N., Iovanna J. (2018). Stratification of Pancreatic Ductal Adenocarcinomas Based on Tumor and Microenvironment Features. Gastroenterology.

[61] Irizarry R.A., Hobbs B., Collin F., Beazer-Barclay Y.D., Antonellis K.J., Scherf U., Speed T.P. (2003). Exploration, normalization, and summaries of high density oligonucleotide array probe level data. Biostatistics.

[62] Chen D.T., Davis-Yadley A.H., Huang P.Y., Husain K., Centeno B.A., Permuth-Wey J., Pimiento J.M., Malafa M. (2015). Prognostic Fifteen-Gene Signature for Early Stage Pancreatic Ductal Adenocarcinoma. PLoS ONE.

[63] Yang S., He P., Wang J., Schetter A., Tang W., Funamizu N., Yanaga K., Uwagawa T., Satoskar A.R., Gaedcke J. (2016). A Novel MIF Signaling Pathway Drives the Malignant Character of Pancreatic Cancer by Targeting NR3C2. Cancer Res..

[64] Winter C., Kristiansen G., Kersting S., Roy J., Aust D., Knosel T., Rummele P., Jahnke B., Hentrich V., Ruckert F. (2012). Google goes cancer: Improving outcome prediction for cancer patients by network-based ranking of marker genes. PLoS Comput. Biol..

[65] Kirby M.K., Ramaker R.C., Gertz J., Davis N.S., Johnston B.E., Oliver P.G., Sexton K.C., Greeno E.W., Christein J.D., Heslin M.J. (2016). RNA sequencing of pancreatic adenocarcinoma tumors yields novel expression patterns associated with long-term survival and reveals a role for ANGPTL4. Mol. Oncol..

[66] Simon R., Lam A., Li M.C., Ngan M., Menenzes S., Zhao Y. (2007). Analysis of gene expression data using BRB-ArrayTools. Cancer Inform..

[67] Tibshirani R. (1997). The lasso method for variable selection in the Cox model. Stat. Med..

[68] Radmacher M.D., McShane L.M., Simon R. (2002). A paradigm for class prediction using gene expression profiles. J. Comput Biol..

[69] Szklarczyk D., Gable A.L., Lyon D., Junge A., Wyder S., Huerta-Cepas J., Simonovic M., Doncheva N.T., Morris J.H., Bork P. (2019). STRING v11: Protein-protein association networks with increased coverage, supporting functional discovery in genome-wide experimental datasets. Nucleic Acids Res..

